# Supplementary material for: A Targeted Epigenetic Clock for the Prediction of Biological Age
Source: Cells. 2022 Dec 14;11(24):4044. doi: 10.3390/cells11244044 (PMC9777448; doi:10.3390/cells11244044)
Supplement: Supplementary file 1 [file cells-11-04044-s001.zip › Supplementary_files/Gensous_Supplementary tables.docx]

**Supplementary Table S1.** Datasets used to select the Infinium CpG probes to be included in the targeted epigenetic assay.

| **Dataset** | **Group** | **N** | **Age range (mean ± SD)** |
| --- | --- | --- | --- |
| Down syndrome Infinium 450k | DS | 29 | 10-43 years  (25.62 ± 9.27) |
|  | DSS | 29 | 9-52 years  (29.83 ± 10.90) |
|  | DSM | 29 | 42-83 years  (58.97 ± 10.41) |
| Long-lived individuals Infinium EPIC | Centenarians | 28 | 101–112  (106.3 ± 2.73) |
|  | Centenarians’ offspring | 19 | 55-89  (71.42 ± 9.29) |
|  | Controls | 30 | 55-82  (71.91 ± 6.62) |

**Supplementary Table S2.** Datasets used to select the Infinium CpG probes to be included in the targeted epigenetic assay.

| **Amplicon ID** | **Forward primer** | **Reverse primer** | **Genomic Localization** |
| --- | --- | --- | --- |
| *ELOVL2* | AGGAAGAGAGGTAAATTTGTAGGAATAGAGTTATTTTTTT | CAGTAATACGACTCACTATAGGGAGAAGGCTCCCCTCTCCCACAAAAACC | chr6:11,044,680-11,045,053 |
| *NHLRC1* | TTGAGTTTAGGAGTTTTATGAGGTG | AACAAAAAACAATCCTATTATCCTCA | chr6:18,122,552-18,123,149 |
| *SIRT7/MAFG* | GAGGGAGGTAGTAGGGATAATATGG | CTTTAACCAAAACCAAATCTCTCAA | chr17:79,877,158-79,877,497 |
| *AIM2* | AAAATTTGGTTGATTGTTGATTTTT | CAATACAAATTCTTATCTTCAAAACA | chr1:159,046,805-159,047,299 |
| *EDARADD* | TTTTTTGGTGATTAGGAGTTTTAGTG | CAAAATTTCAAAAAACAAACCAACT | chr1:236,557,384-236,557,805 |
| *TFAP2E* | TTATTATAATTGGAGTGTATGGAGTAGG | ACAAAAAAATTAAAAAATCCAACAC | chr1:36,038,876-36,039,325 |
