# Supplementary material for: A Targeted Epigenetic Clock for the Prediction of Biological Age
Source: Cells. 2022 Dec 14;11(24):4044. doi: 10.3390/cells11244044 (PMC9777448; doi:10.3390/cells11244044)
Supplement: Supplementary file 1 [file cells-11-04044-s001.zip › Supplementary_files/Gensous_Supplementary tables.pdf]

Supplementary Table S1. Datasets used to select the Infinium CpG probes to be included in the targeted epigenetic assay.

Dataset	Group	N	Age range (mean ± SD)
Down syndrome Infinium 450k	DS	29	10-43 years (25.62 ± 9.27)
	DSS	29	9-52 years (29.83 ± 10.90)
	DSM	29	42-83 years (58.97 ± 10.41)
Long-lived individuals Infinium EPIC	Centenarians	28	101–112 (106.3 ± 2.73)
	Centenarians' offspring	19	55-89 (71.42 ± 9.29)
	Controls	30	55-82 (71.91 ± 6.62)

Supplementary Table S2. Datasets used to select the Infinium CpG probes to be included in the targeted epigenetic assay.

Amplicon ID	Forward primer	Reverse primer	Genomic Localization
ELOVL2	AGGAAGAGAGGTAAATTTGTAGGAATAGAGTTATTTTTT	CAGTAATACGACTCACTATAGGGAGAAGGCTCCCCTCTCCCACAAAAACC	chr6:11,044,680-11,045,053
NHLRC1	TTGAGTTTAGGAGTTTTATGAGGTG	AACAAAAAACAATCCTATTATCCTCA	chr6:18,122,552-18,123,149
SIRT7/MAFG	GAGGGAGGTAGTAGGGATACATATGG	TTTAACCAAAACCAAATCTCTCAA	chr17:79,877,158-79,877,497
AIM2	AAAATTTGGTTGATTGTTGATTTTT	CAATACAAATTCTTATCTTCAAAACA	chr1:159,046,805-159,047,299
EDARADD	TTTTTTGGTGATTAGGAGTTTTAGTG	CAAAATTTCAAAAAACAAACCAACT	chr1:236,557,384-236,557,805
TFAP2E	TTATTATAATTGGAGTGTATGGAGTAGG	ACAAAAAAATTAAAAAATCCAACAC	chr1:36,038,876-36,039,325
