# Supplementary material for: A Targeted Epigenetic Clock for the Prediction of Biological Age
Source: Cells. 2022 Dec 14;11(24):4044. doi: 10.3390/cells11244044 (PMC9777448; doi:10.3390/cells11244044)

Genomic region	CpG units
ELOVL2	CpG_2.3, CpG_5, CpG_6.7, CpG_8, CpG_9, CpG_10, CpG_11.12.13.14, CpG_15.16.17, CpG_18.19.20.21, CpG_22.23.24, CpG_27, CpG_28.29, CpG_30.31.32, CpG_33, CpG_34.35.36
NHLRC1	CpG_1, CpG_2.3, CpG_4, CpG_5, CpG_6, CpG_7.8.9, CpG_10, CpG_12.13, CpG_20.21.22, CpG_23.24.25, CpG_26.27, CpG_28.29, CpG_36.37, CpG_39, CpG_40.41, CpG_42.43, CpG_44.45.46, CpG_48.49.50, CpG_51, CpG_52.53, CpG_54
SIRT7	CpG_1, CpG_2, CpG_3.4, CpG_5, CpG_6, CpG_7
AIM2	CpG_1, CpG_2, CpG_3, CpG_5, CpG_6, CpG_7, CpG_8
EDARADD	CpG_1, CpG_2, CpG_3, CpG_4, CpG_6.7
TFAP2E	CpG_1, CpG_2.3, CpG_4, CpG_6, CpG_9.10, CpG_11, CpG_12, CpG_14.15, CpG_16, CpG_24.25.26.27, CpG_28.29.30, CpG_31.32.33.34, CpG_35, CpG_37.38, CpG_39.40, CpG_41.42

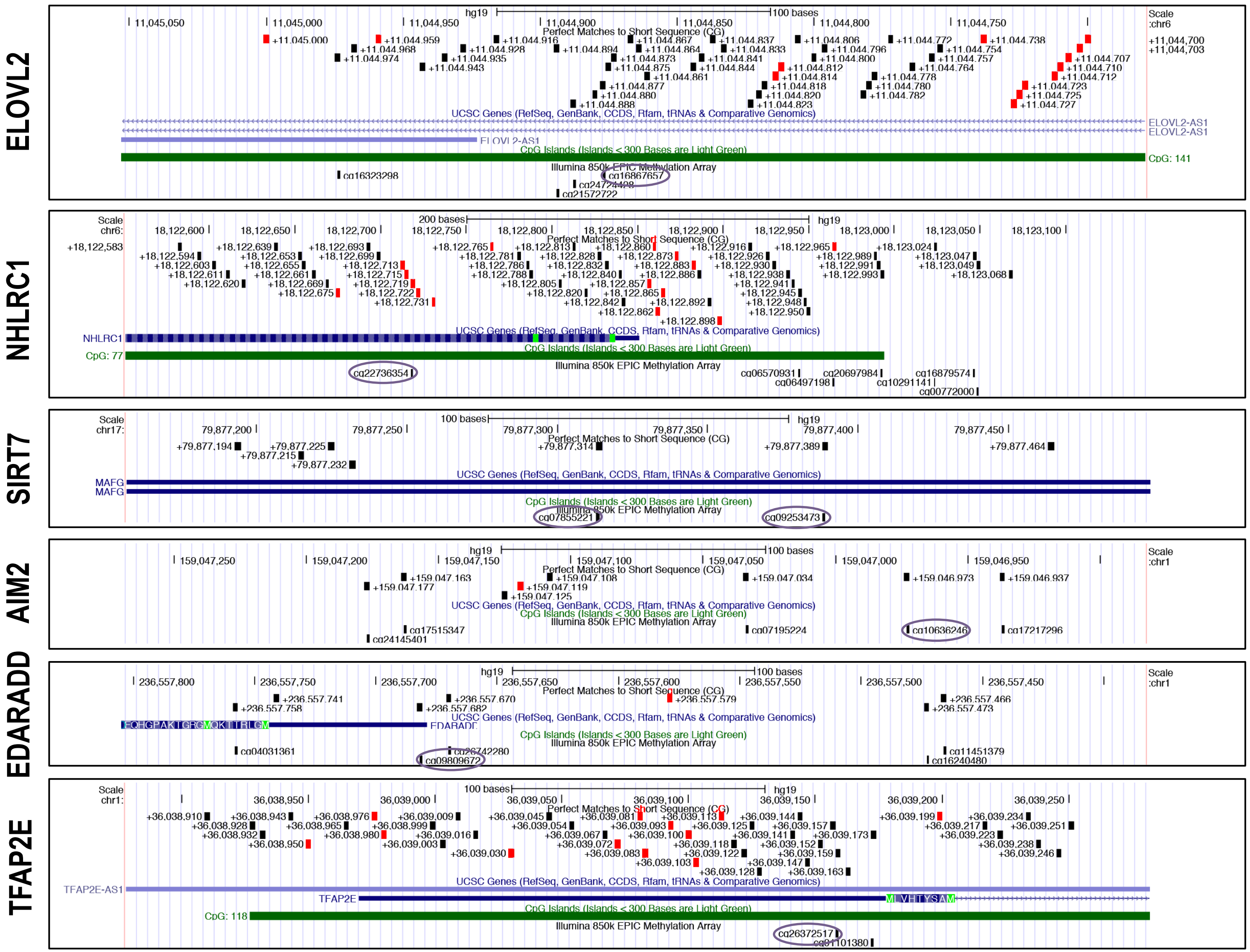

Supplement: Supplementary file 1 [file cells-11-04044-s001.zip › Supplementary_files/Supplementary Figure S1.pdf]
