# Supplementary material for: A Targeted Epigenetic Clock for the Prediction of Biological Age
Source: Cells. 2022 Dec 14;11(24):4044. doi: 10.3390/cells11244044 (PMC9777448; doi:10.3390/cells11244044)
Supplement: Supplementary file 1 [file cells-11-04044-s001.zip › Supplementary_files/Supplementary_Figure S4.pdf]

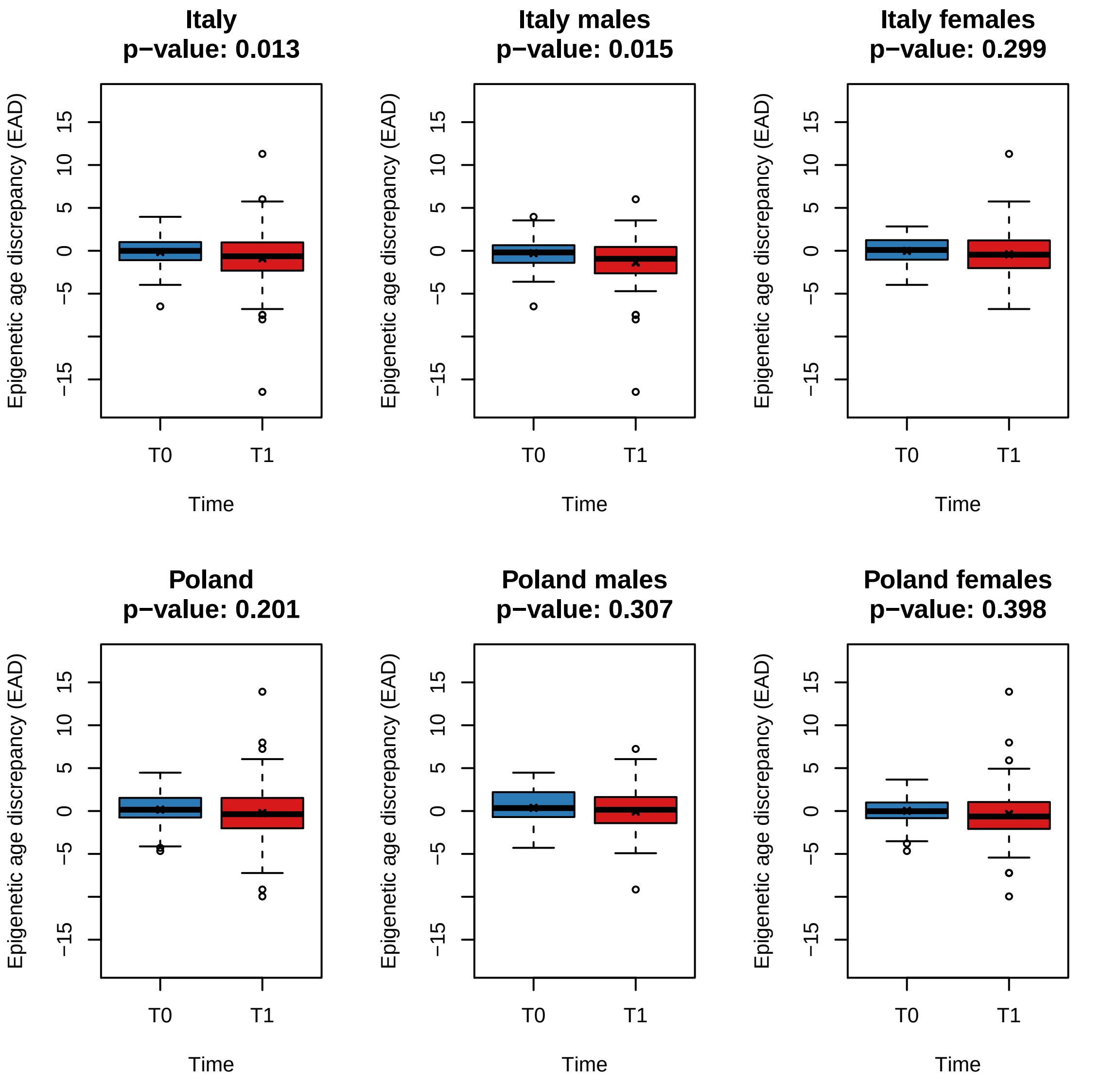
Italy
p−value: 0.013
Italy males
p−value: 0.015
Italy females
p−value: 0.299
Poland
p−value: 0.201
Poland males
p−value: 0.307
Poland females
p−value: 0.398
Epigenetic age discrepancy (EAD)
Time
T0
T1
15
10
5
0
−5
−15
