# Supplementary figures and images for: A Targeted Epigenetic Clock for the Prediction of Biological Age
Source: Cells. 2022 Dec 14;11(24):4044. doi: 10.3390/cells11244044 (PMC9777448; doi:10.3390/cells11244044)

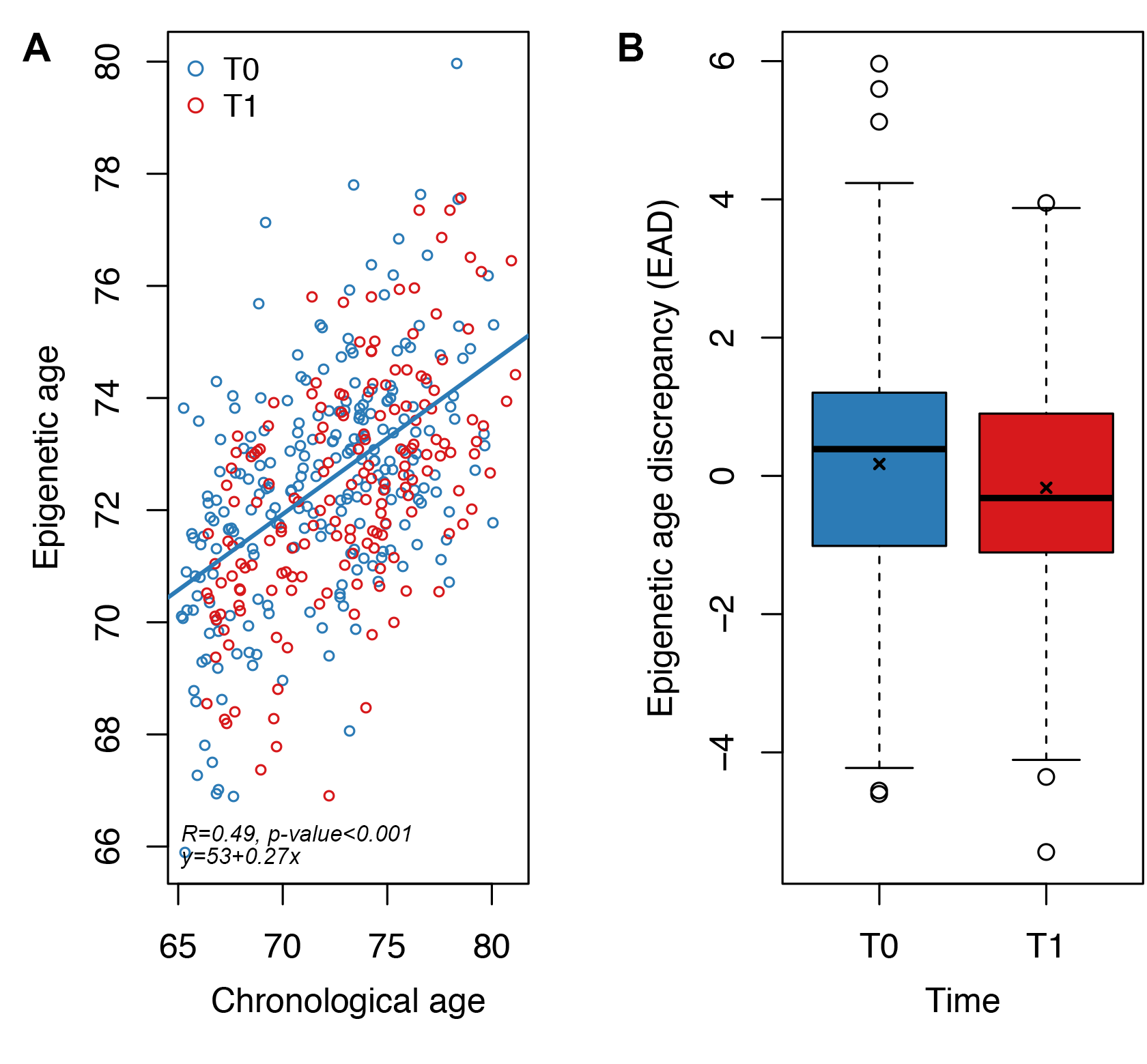

Supplement: Supplementary file 1 [file cells-11-04044-s001.zip › Supplementary_files/Supplementary_Figure S2.png]

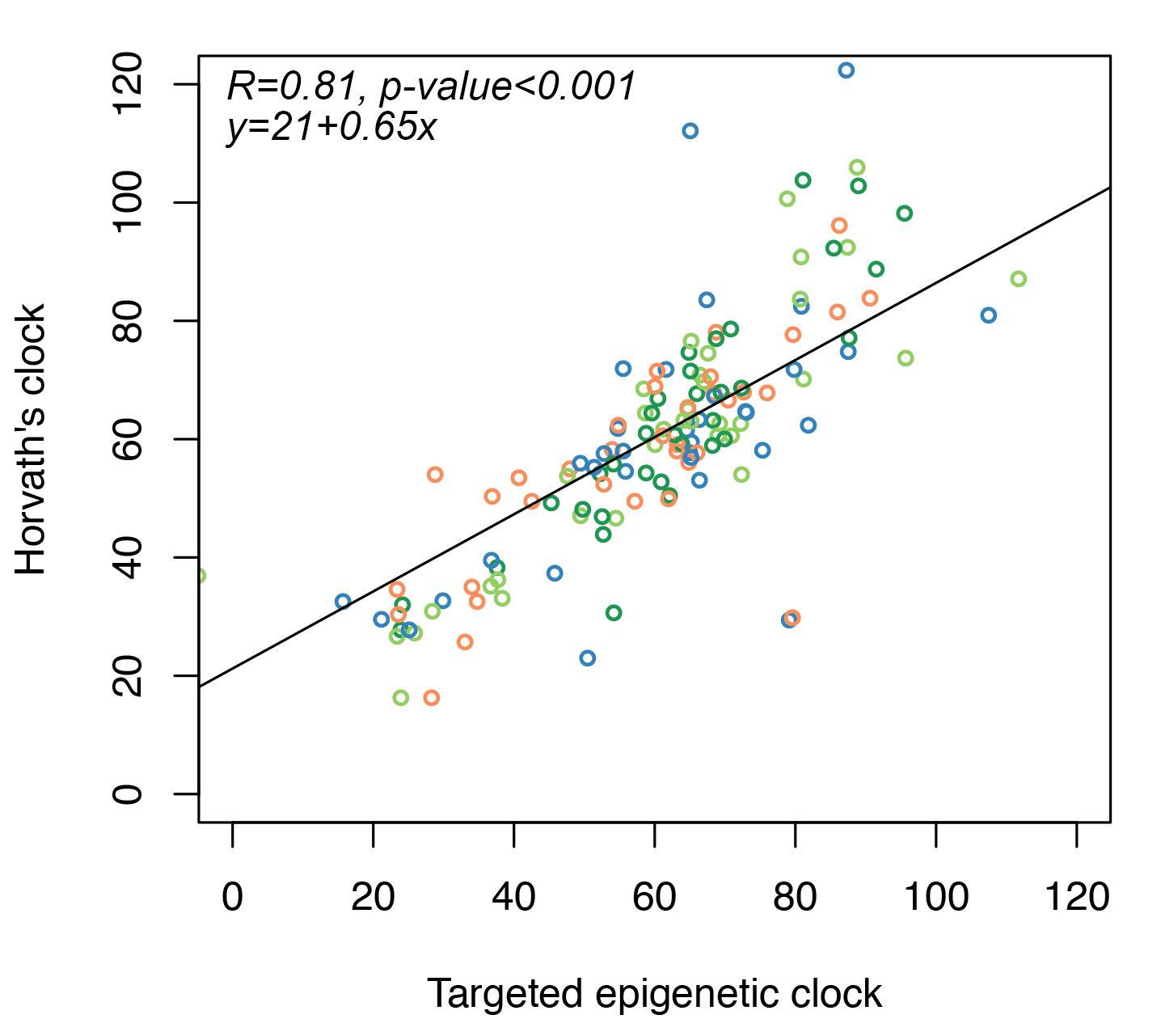

Supplement: Supplementary file 1 [file cells-11-04044-s001.zip › Supplementary_files/Supplementary_Figure S3.png]
